# Graphene oxide membrane chemically modified by electron-transfer diazonium chemistry for efficient dye separation†

**DOI:** 10.1039/d2ra03886b

**Published:** 2022-10-19

**Authors:** Zhenjiang Li, Yucheng Xing, Yan Liu, Alan Meng, Xiaoyan Fan

**Affiliations:** College of Materials Science and Engineering, Qingdao University of Science and Technology Qingdao 266061 Shandong P. R. China; School of Mathematics and Physics, Qingdao University of Science and Technology Qingdao 266061 Shandong P. R. China lzfxy@126.com; College of Chemistry and Molecular Engineering, Qingdao University of Science and Technology Qingdao 266061 Shandong P. R. China

## Abstract

By chemical modification of the graphene oxide (GO) surface *via* diazonium chemistry, we introduce nitrobenzene groups as new interlayer pillars to GO memebranes like the surface oxygen-containing functional groups. The larger pillar can finely enlarge the interlayer space of the GO membrane. The filtration performance of modified GO membranes with different mass ratios of nitrobenzene diazonium tetrafluoroborate (NDT) were tested for EB, DR81, and MB. Notably, when the GO : NDT ratio is 1 : 1, it is found that the water flux can be enhanced by more than twice and by nearly 1.4 times its value for EB and DR81, respectively, while maintaining a high rejection (92% for EB and 95% for DR81). In conclusion, the chemical modification of GO through the dediazonization reaction of NDT can indeed improve the separation efficiency of the dye.

## Abstract 2

By diazonium chemistry, nitrobenzene groups have been introduced to graphene oxide membranes as interlayer pillars like the surface oxygen-containing functional groups.

## Graphical abstract

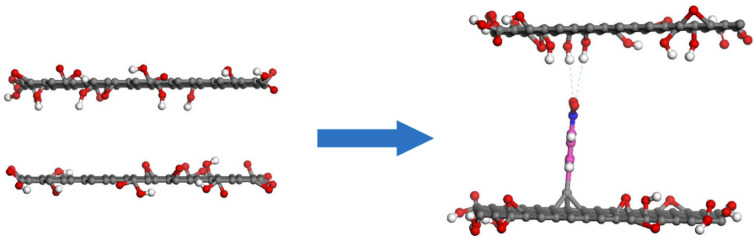

## 1 Introduction

With the development of economy and society, a large amount of industrial dye wastewater is discharged every day. It has characteristics of a high chroma, high toxicity and difficult degradation. Membrane separation technology is an effective way to solve dye pollution through removing dye molecules.^1–5^ The core research of membrane technology are membrane materials.

Graphene oxide (GO) is the oxidized derivative of graphene. Besides its excellent mechanical properties, large specific surface area and stable chemical properties, many polar oxygen-containing functional groups are distributed on the surface and edge of GO, which enables good hydrophilicity and the ability to be well dispersed in water, thus providing great convenience for the preparation of graphene oxide membranes.^6^ Through a simple assembly method, GO membranes can be prepared on a large scale. Therefore, GO is an excellent two-dimensional material for constructing filtration membranes and has potential application in the field of membrane separation.

GO can be regarded as being composed of sp^2^ nanoclusters (non-oxidized region) and sp^3^ oxygen-containing functional groups (oxidized region). A layered graphene oxide separation membrane is formed by stacking single-layer atomic thickness GO sheets in close parallel by vacuum suction filtration.^7^ Due to the strong hydrogen bonding, the oxygen-containing functional groups can act as interlayer pillars to form two-dimensional nanochannels between GO interlayers. The channel size in aqueous solution is about ∼1 nm. Solutes, the hydrated radii of >0.45 nm, can be sieved out by the physical size effect of the nanochannels within GO membranes.^8^ It has a high injection capability for larger dye molecules. However, the filtration efficiency (the water flux) is constrained due to the smaller channel size relative to that of the larger dye molecules. The channel size can be finely enlarged to improve the filtration efficiency. Moreover, theoretical calculation studies indicate that enlarging the interlayer space can improve the flux of the GO membrane.^9^ One enlargement strategy is the intercalation of other low-dimensional materials, such as nanoparticles,^10^ nanotubes,^11^ nanowires,^12^ and nanoflakes.^13,14^ The other strategy is chemical crosslinking by organic and inorganic species.^15,16^ According to previous reports, graphene oxide can be covalently modified by diazonium chemistry.^17,18^ The aryl group can be grafted on the surface of graphene oxide. The size of aryl groups is larger than the oxygen-containing functional groups distributed on the surface of graphene oxide. Therefore, it can be expected that the interlayer space will be increased. Subsequently, the water flux will increase with the rejection ratio of the larger dye molecule being maintained if the interlayer space size is smaller than the size of the dye molecule.

In this paper, the surface of the single GO is chemically modified by nitrodiazonium. The chemically modified GO membrane is fabricated by vacuum filtration. It shows a high separation efficiency of larger dye molecules.

## 2 Experimental section

### 2.1 Materials

Graphite, Evans blue and Methyl blue were purchased from Sinopharm Chemical Reagent Co., Ltd. Potassium permanganate (KMnO_4_, A.R. grade), nitric acid (HNO_3_, A.R. grade), hydrochloric acid (HCl, A.R. grade) and hydrogen peroxide (H_2_O_2_, A.R. grade) were purchased from Yantai Sanhe Chemical Co., Ltd. Direct red 81 (DR81) was purchased from TCI (Shanghai) Development Co., Ltd. Rhodamine B was purchased from Tianjin Dengke Chemical Co., Ltd. 4-Nitrobenzene diazonium tetrafluoroborate (NDT) was purchased from Sigma-Aldrich. Mixed cellulose ester membranes (MCE, 50 mm in diameter), as supporting membranes with a pore size of 0.22 μm, were provided by Shanghai Xingya material works.

### 2.2 Preparation of GO membranes

GO flakes were synthesized according to an improved Hummers' method.^19^ Briefly, graphite was exposed to a mixture of concentrated H_2_SO_4_, H_3_PO_4_ and KMnO_4_. Excessive oxidizing agents were removed using H_2_O_2_ and HCl, followed by multiple washing. The sediment was dried at 50 °C for 24 h. The XRD and TEM measurements of the as-prepared powder are shown in Fig. S1,† indicating that GO single layer flakes with a size of hundreds of nanometres were successfully prepared.

Three millilitres of a GO dispersion with a concentration of 0.2 mg ml^−1^ were added to the flask, followed by the addition of different volumes of NDT with a concentration of 10 mmoL L^−1^. The mixture was diluted by deionized water to 50 ml, then stirred at 37 °C for 1 h. The dispersion was sonicated for 10 min and then filtrated on MCE membranes (0.22 μm in pore diameter, 47 mm in diameter) under vacuum by a homemade separation device (Fig. S2†). The as-prepared GO membranes were dried at 50 °C for 6 h before further use.

### 2.3 Performance evaluation

The dye separation performance of GO membranes was evaluated by filtrating EB, DR81 and MB on a filtration device with an effective area of 2.14 cm^2^, driven at 1 bar. The retention of dyes is calculated by the following equation:1
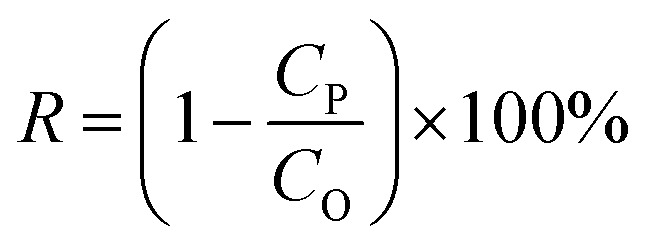
where *C*_P_ and *C*_O_ are the concentration of the collected permeate and feed solution, respectively. The water flux is calculated as:2
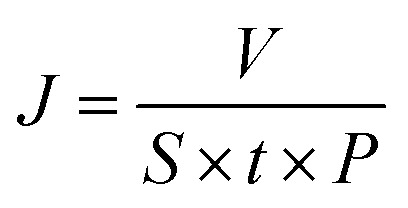
where *V*, *S*, *t*, *P* are the volume of permeate solution, the effective area of the membrane, filtration time, and operation pressure, respectively.

### 2.4 Characterization

X-ray diffraction (XRD) spectra of the as-prepared membranes was carried out on an X-ray diffractometer (D8 ADVANCE, Germany) with Cu *K*_α_ radiation (*λ* = 0.15406 nm). The morphologies were visualized by scanning electron microscopy (SEM; JSM-7500F, Japan). Raman spectroscopy (Raman Microscope DXR, America) was performed to characterize the typical structural features of GO membranes. X-ray photoelectron spectroscopy (XPS) (Thermo ESCALAB 250Xi, America) and Fourier transform infrared spectroscopy (FTIR) (Thermo iS20, America) were employed to detect the variation of element content. UV-vis spectra were acquired by a UV-vis spectrophotometer (Lambda 35 PerkinElmer, USA).

## 3 Results and discussion

### 3.1 Characterization of GO membranes

**Fig. 1:**
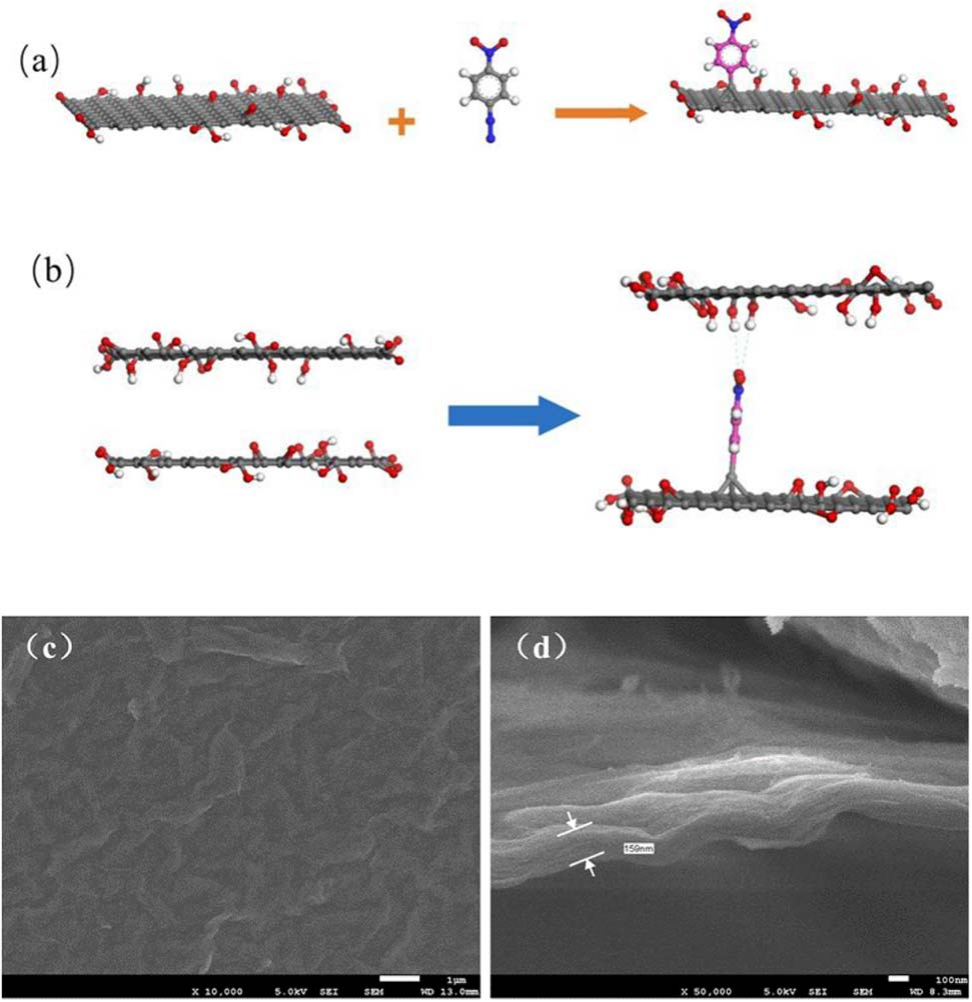
(a) Mechanism of the aryl group grafting process; (b) mechanism of the interlayer channel enlargement; SEM images of the (c) surface and (d) cross-section of GO membranes modified by NDT.

In the modification process, the nitrobenzene diazonium ion is reduced to a nitrobenzene (NB) radical through electron-transfer from GO. The radical subsequently grafts on the surface of GO by C–C covalent bonding which introduces the change in hybridization of carbon from sp^2^ to sp^3^ on the edges and surface (Fig. 1(a)).^18^ The nitro group at the para position tends to exist in a chelating bidentate configuration with O-containing groups/edges/defects and the hydrogen of H_2_O *via* electrostatic interactions and polarization,^20^ as shown in Fig. 1(b). This can maintain the stable layered structure of the GO membrane in aqueous solution like cross-linking effect.

Fig. 1(c) and (d) present the SEM images of GO membranes modified by NDT in a 1 : 1 ratio of GO : NDT. The acquired modified GO membranes were uniformly and densely laminated on porous support membranes and the thickness of the modified GO membrane is approximately 160 nm. This indicates that the modification has not induced the collapse of interlayer channels. Moreover, the surface of the GO membrane is flat and no flaws are discovered.

**Fig. 2:**
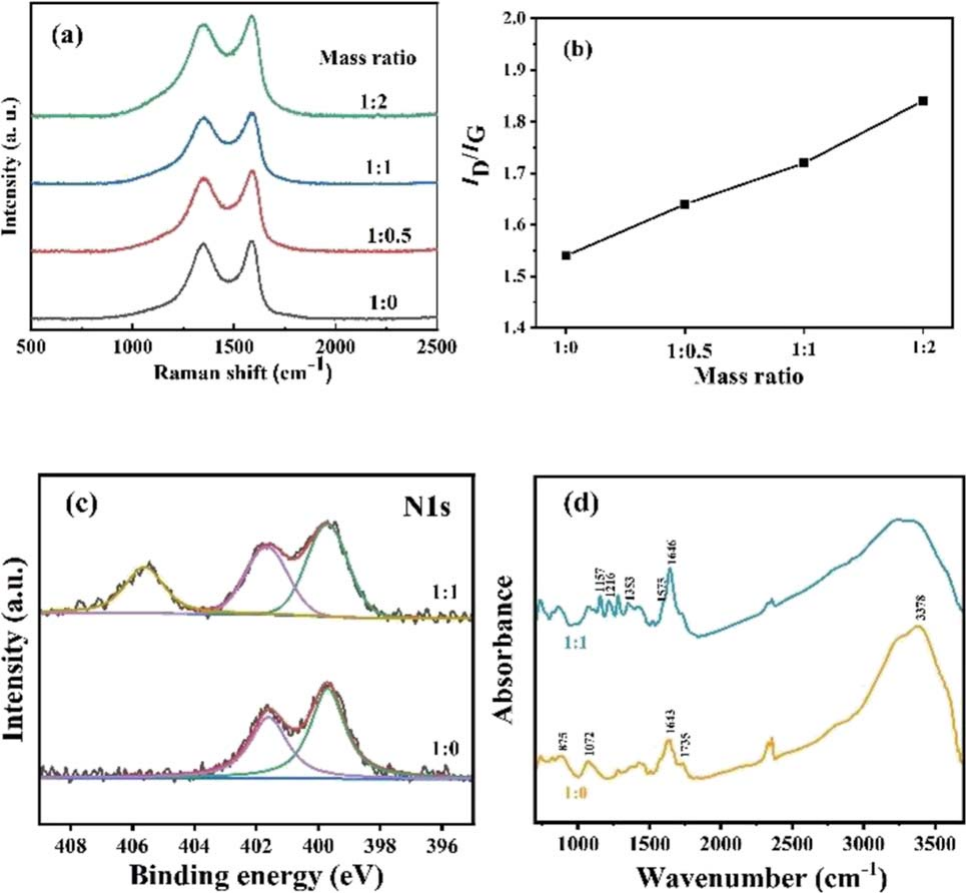
(a) Raman spectra and (b) *I*_D_/*I*_G_ ratio of GO membranes with different ratios of GO : NDT; (c) XPS of N 1s; (d) FTIR spectra of GO membranes.

Raman spectra were introduced to confirm the chemical attachment of NB groups of GO membranes. Fig. 2(a) presents the detailed Raman spectra of GO membranes modified by NDT with different ratios of GO to NDT. Two typical peaks appear in the spectra. The peak located at 1350 cm^−1^ is the D band, which is ascribed to structural defects and sp^3^-hydridized bonds; the peak located at 1585 cm^−1^ is the G band, which arises from the *E*_2g_ phonon of C sp^2^ atoms.^21^ The integrated intensity ratio of D and G bands, namely *I*_D_/*I*_G_, usually quantifies the disorder in the carbon materials. As can be seen in Fig. 2(b), the *I*_D_/*I*_G_ values of GO membranes with modifying ratios 1 : 0, 1 : 0.5, 1 : 1 and 1 : 2 of GO/NDT are 1.54, 1.64, 1.72 and 1.84, respectively, which indicates that the defects of GO increase with the increase of NDT ratios. It results from a greater disorder induced by C–C covalent bonding between NB and GO. Therefore, the *I*_D_/*I*_G_ value increases with the increase of NDT ratios, which verified that chemical modification occurred.

XPS and FTIR analysis were further used to confirm the targeted chemical modification. The survey scan of the GO membranes (Fig. S3a†) reveals strong C 1s peaks and O 1s peaks accompanied with weak N1s peaks. In Fig. S3b,† deconvolution of the C 1s spectrum for the pure GO membrane is carried out using four bands at 284.80, 286.75, 287.30, and 288.80 eV, which are assigned to C=C, C–OH, C–O–C, and C=O (carbonyl and carboxyl) bonds, respectively.^22^ After the chemical attachment of NB groups, the peak at 284.80 eV becomes broader and deconvolution reveals that the peak with a bonding energy (BE) of 285.00 eV is assigned to C–N of NB groups.^23^ N 1s peaks for the pure GO membrane show two peaks located at 399.70 and 401.65 eV, which originate from the chemical reagent used in the GO preparation process. After NB chemical attachment, a higher BE peak at 405.60 eV appears, which is attributed to the nitro groups and confirms the presence of nitrophenyl groups on the GO surface (Fig. 2(c)).^23^ The high solution of O1s peaks results from oxygen groups (C–OH, C–O–C, C=O and O=C–OH). After NB chemical attachment, the peak is broader which is due to the introduction of O–N bonds in NB groups (Fig. S3c).† In Fig. 2(d), the IR spectrum of the GO membrane without modification shows typical features. The broad band at around 3378 cm^−1^ is assigned to O–H stretching vibrations of hydroxyl groups and adsorbed water on the surface of the GO membranes.^18^ The band at 1735 cm^−1^ is the stretching mode of carboxyl groups.^24^ The band at 1643 cm^−1^ corresponds to unoxidized C=C stretching vibrations of benzene rings and NB groups.^24^ The band at 1072 cm^−1^ and 875 cm^−1^ is attributed to the C–O stretching mode and epoxy stretching mode.^24^ The IR spectrum of the modified GO membrane with an GO : NDT ratio of 1 : 1 shows that the band of symmetric and anti-symmetric vibrations of the –NO_2_ group are located at 1353 cm^−1^ and 1575 cm^−1^, respectively.^23,25^ The C–N stretch and C–H in-plane bending vibrations of NB groups are located at 1157 cm^−1^ and 1216 cm^−1^, respectively.

**Fig. 3:**
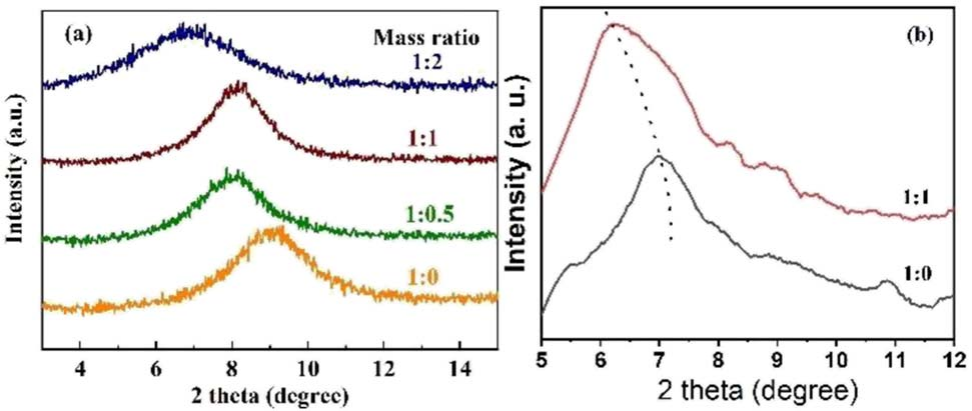
XRD patterns of modified GO membranes with different ratios of GO : NDT: (a) dry state; (b) wet state.

The interlayer spacing of modified GO membranes with different ratios of GO : NDT was investigated by X-ray diffraction, as shown in Fig. S4† and 3. Only one obvious peak appears for all membranes with different mass ratios. The diffraction peak of the pure GO membrane is located at 2*θ* = 9.26°, corresponding to an interlayer spacing of *d* = 0.95 nm. After being modified by NTD, the diffraction angles of GO membranes tend to become larger, corresponding to an interlayer spacing of *d* = 1.09, 1.09 and 1.28 nm for GO membranes modified by mass ratios 1 : 0.5, 1 : 1 and 1 : 2, respectively. To investigate the swelling effect in the fully wet state, XRD patterns are also presented in Fig. 3(b). It is obvious that the diffraction peaks of the modified GO membranes shift to lower angles compared with the dry state membranes, indicating that the interlayer spacing is enlarged. The increase in d-spacing is attributed to the damage of the hydrogen bonds after the water molecules enter the GO layers.^26^ Moreover, due to hydrolysis of the carboxyl groups in the fully wet state, the GO sheets are negatively charged and repel each other due to the electrostatic repulsion, giving rise to a large spacing. The peaks of the pure GO membrane and modified GO membrane with a mass ratio of 1 : 1 shift to 7.02° and 6.22°, respectively. Correspondingly, the d-spacings increase to 12.58 and 14.20 nm, respectively. This indicates that the interlayer size of the GO membrane in water can be further increased due to the further weakening of hydrogen bonds of the interlayer induced by the chemical attachment of NB groups compared with the pure GO membrane. Therefore, the interlayer is, indeed, finely broadened, which is as expected after modification by NTD.

### 3.2 Separation performance of GO membranes

**Fig. 4:**
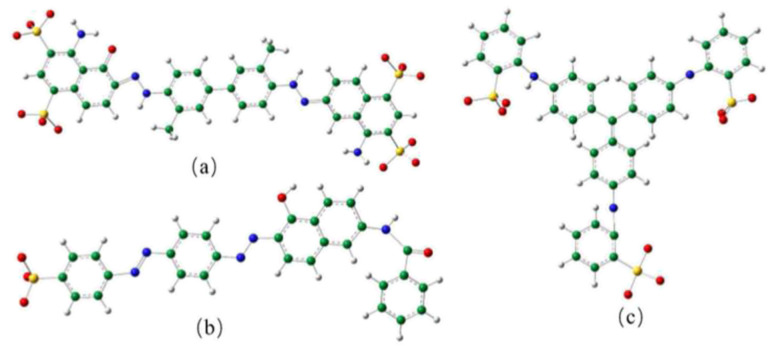
Structure of the (a) EB molecule, (b) DR81 molecule, and (c) MB molecule.

The separation performance of the modified GO membranes to negatively charged EB (2.7 nm × 0.8 nm), DR81 (2.5 nm × 0.8 nm), and MB (1.9 nm) was evaluated. The structures of the three dye molecules are shown in Fig. 4.

**Fig. 5:**
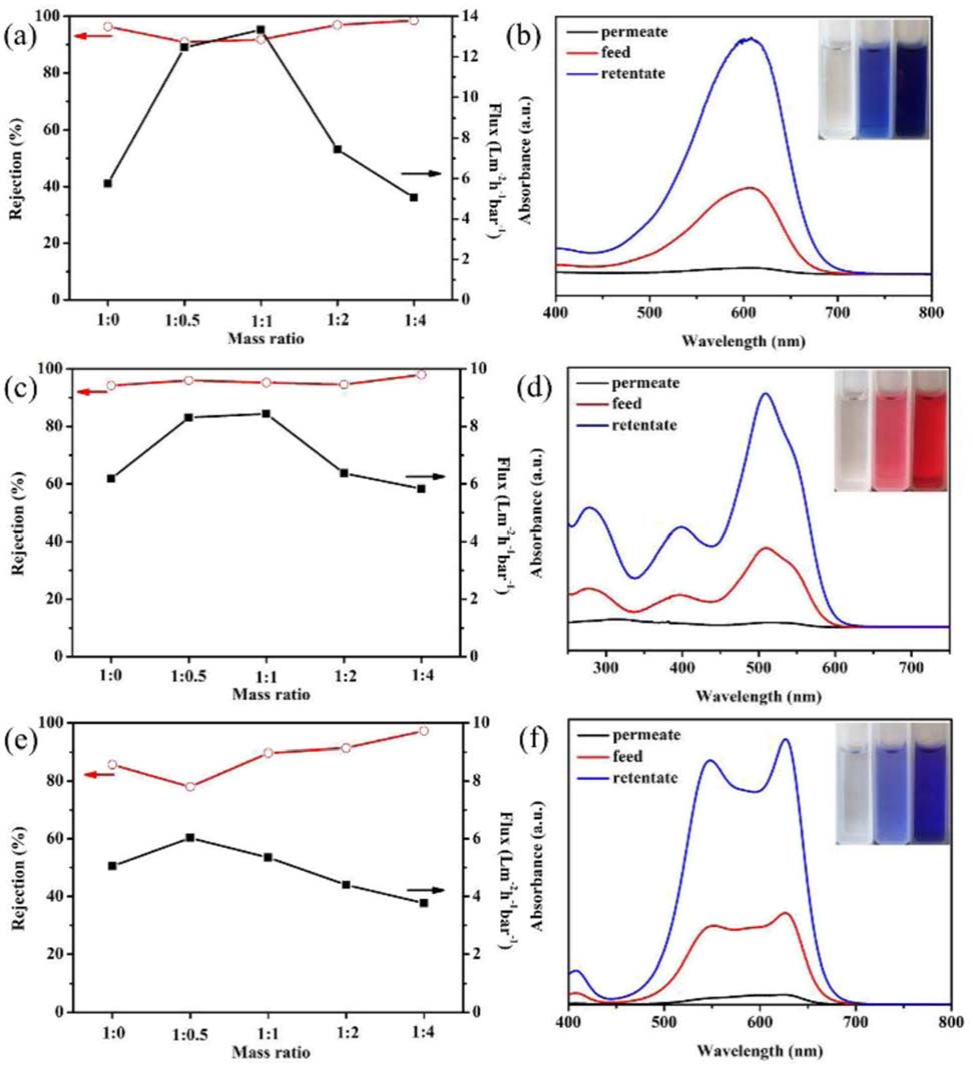
(a), (c) and (e) Separation performance of GO membranes modified by different ratios of NDT for EB, DR81, and MB, respectively. (b), (d) and (f) Absorbance of the permeate, feed, and retentate solution of EB, DR81, and MB, respectively.

Fig. 5(a) presents the EB separation performance of GO membranes modified by different ratios of GO : NDT. The pure GO membrane can remove most of the EB molecules, and the rejection is 96.4%. However, the water flux is only 5.75 L m^−2^ h^−1^ bar^−1^, which is rather low. When the mass ratio of GO : NDT is 1 : 0.5, the water flux increases to 12.46 L m^−2^ h^−1^ bar^−1^. The flux reaches a maximum value of 13.34 L m^−2^ h^−1^ bar^−1^ at a GO : NDT mass ratio of 1 : 1, which is more than twice the value of the pure GO membrane. The rejection is kept at above 90%. The enhancement of the flux is reasonably attributed to the broadening of the interlayer spacing through the covalent grafting of NB on the non-oxidized region of the GO surface, which is verified by the above results. Thus, it can be concluded that expanding the interlayer space is an efficient way to improve the flux of the GO membrane. Further, the flux gradually decreases with the increase of the NDT ratio, and is slightly lower than that of the pure GO membrane when the ratio reaches 1 : 4. The corresponding rejection is improved to 99%. GO consists of the oxidized regions and pristine graphene. The oxidized regions are full of oxygen-containing functional groups. Water molecules flow rather slowly due to the interaction between water molecules and oxygen-containing functional groups, while water molecules can transport unimpededly in the pristine graphene due to the capillary phenomenon.^7^ When the ratio of NDT increases, the pristine region composed of C=C bonds is almost destroyed, resulting in the significant reduction of the “unimpeded nanochannel”, which contributes to the declined flux. In addition, the unreacted NDT cations will absorb on GO sheets through a chelating configuration with carboxyl and hydroxyl groups with the increasing of the NDT ratio.^18^ The steric effect of unreacted NDT cations and covalently bonded nitrobenzenes existing in the galleries will reduce the flux. Meanwhile, the rejection can be enhanced. Therefore, the filtration efficiency can be effectively enhanced through modification by a certain ratio of NDT without sacrificing the rejection towards EB dyes.

Similar phenomena have also been found in the filtration process of DR81 and MB in Fig. 5(c) and (e). In Fig. 5(c), the flux has been enhanced by 37% maximally when the GO : NDT ratio is 1 : 0.5 and the rejection ratios of DR81 remain above 95% after modification. The higher rejection indicates that the size of the interlayer space is small enough for the filtration of EB and *DR81 molecules. Likewise, the flux of MB increases first and then decreases with the increase of the NDT ratio, as presented in Fig. 5(e). Although the rejection of MB for the modified GO membrane with a GO : NDT ratio of 1 : 0.5 is reduced to 78.0% from 85.6% of pure GO, the reduction is relatively smaller. Therefore, it can be estimated that the average size of the interlayer space of the modified GO membrane in aqueous solution is lower than 1.9 nm, *i.e.* the size of the MB molecule. The increase of the rejection of DR81 and MB with the further increase of the GO/NDT ratio can be also reasonably attributed to the steric effect.

To distinguish whether EB is removed by interception or adsorption, a test was designed. EB aqueous solution with 150 μg of solute, which is defined as the feed solution, was filtrated by the GO membrane with a mass ratio of 1 : 1. After half of the filtrating process was complete, the unfiltered half of the EB solution above the membrane, defined as the retentate solution, and the other half of the filtered solution, defined as the permeate solution, were collected. The absorbances of the feed, retentate and permeate solution were measured by UV-vis spectroscopy, as presented in Fig. 5(b). The inset of Fig. 5(b) shows digital images of the feed, retentate and permeate solution. The absorbance peak of the EB permeate solution barely existed. Combined with the inset digital photos, it can be determined that the EB molecules are almost removed. The absorbance peak of the retentate is much higher than that of the feed solution. According to the standard curve (Fig. S5†), the concentration of the solution can be acquired. The calculation by concentration and volume indicates that the total mass of solutes in the permeate and retentate solution is 146.61 μg, which is very close to that of the feed solution, *i.e.* 150 μg. This suggests that the EB molecules are not adsorbed on the membranes, which results from the electrostatic repulsion between the GO membrane and negatively charged EB dyes. The same phenomenon is observed in the filtration process of DR81 and MB, as shown in Fig. 5(d) and (f). The total mass of solutes in the permeate and retentate solution of DR81 and MB is 147.48 and 144.36 μg, respectively, which is also very close to that of the feed solution (150 μg). Therefore, it can be inferred that the removal mechanism of these three types of dye molecules for the modified GO membrane is interception rather than absorption. Based on the above results, it can be concluded that the retention results from the intense interaction of the three negatively charged dye molecules and the negatively charged GO in aqueous solution.

## 4 Conclusions

NB can be anchored on the surface of a GO single layer through the dediazonization process. The NB groups can act as larger pillar-like oxygen-containing groups on the GO surface. The membrane was acquired by vacuum filtration of the modified GO with different GO : NDT ratios. The chemical attachment of NB groups was confirmed by Raman, XPS and FTIR spectra. The XRD results indicate that the interlayer space can indeed be finely enlarged by the modification. Morphology studies indicate that both the surface and the stacking of the modified GO are uniform. The modified GO membranes exhibit enhanced flux without sacrificing the high rejection of dyes, especially for EB and DR81. The flux can be improved by more than twice and by nearly 1.4 times its value compared to that of the pure GO membrane, while maintaining a high rejection of above 90% for EB and 95% for DR81, respectively. Moreover, the effective separation diameter of the GO membranes is speculated to be smaller than 1.9 nm in aqueous solution. In conclusion, the chemical modification of GO through a dediazonization reaction of NDT can indeed enlarge the interlayer space size, and subsequently improve the separation efficiency of the dyes.

## Author contributions

Project conceptualization, methodology, and supervision, Zhenjiang Li and Xioayan Fan; administration and funding acquisition, Zhenjiang Li and Xiaoyan Fan; methodology, investigation, data analysis, and writing – original draft preparation, Xiaoyan Fan, Zhenjiang Li, Yucheng Xing, Yan Liu, and Alan Meng; writing – review and editing, Xiayan Fan. All authors contributed to the discussion and approval of the manuscript for publication.

## Conflicts of interest

There are no conflicts to declare.

## Supplementary Material

RA-012-D2RA03886B-s001
