# Supplementary material for: Graphene oxide membrane chemically modified by electron-transfer diazonium chemistry for efficient dye separation
Source: RSC Adv. 2022 Oct 19;12(46):29878–83. doi: 10.1039/d2ra03886b (PMC9580474; doi:10.1039/d2ra03886b)
Supplement: RA-012-D2RA03886B-s001 [file RA-012-D2RA03886B-s001.pdf]

Electronic Supplementary Material (ESI) for RSC Advances.
This journal is © The Royal Society of Chemistry 2022

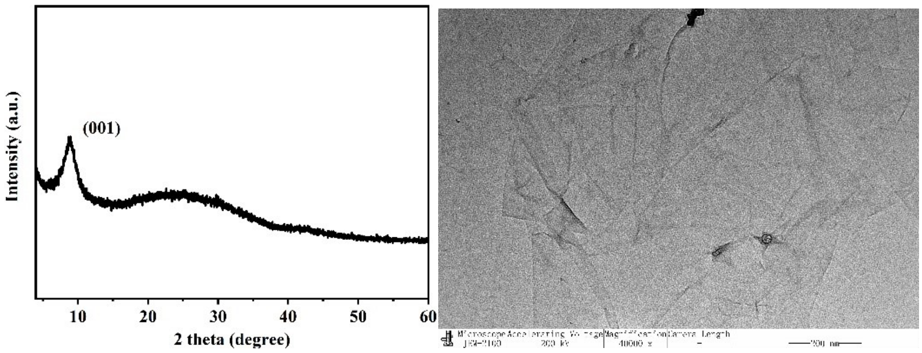

Fig. S1 XRD and TEM images of the as-prepared GO sheets

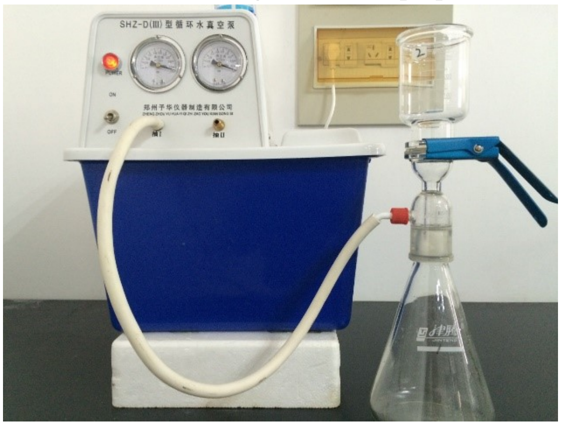

Fig. S2 Homemade separation device

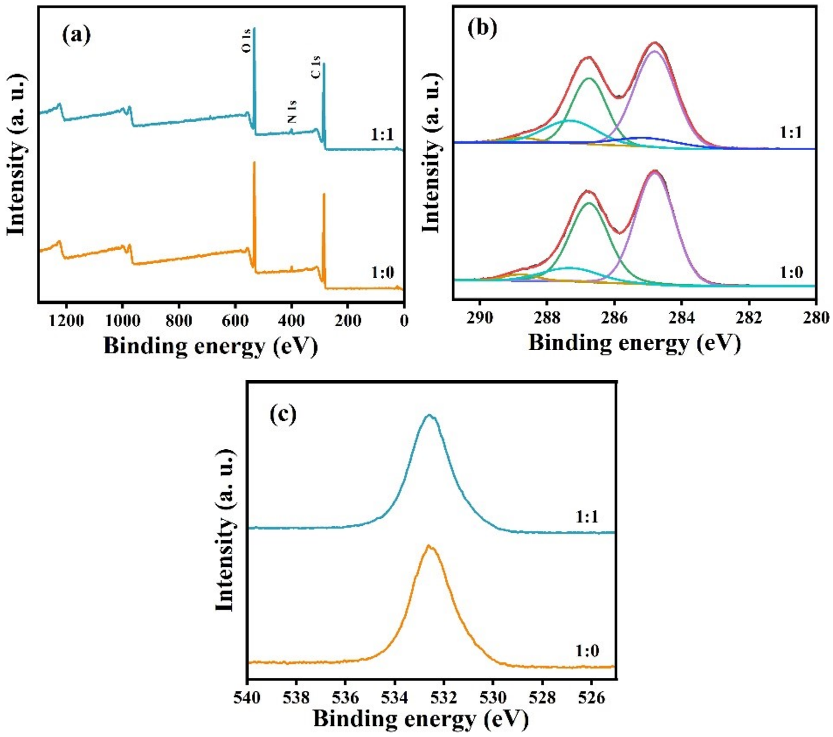

Fig.S3 XPS spectra of modified GO membranes: (a) full spectra;
(b) C1s core level spectra; (c) O1s C1s core level spectra.

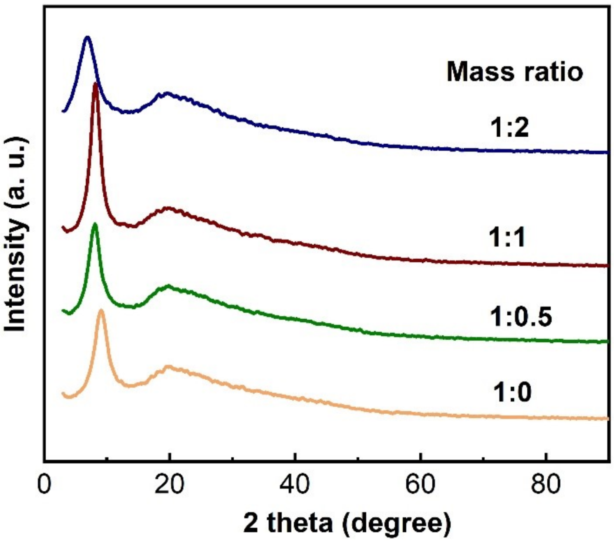

Fig.S4 XRD patterns in an intensive range of modified GO membranes with different ratios of GO to NDT.

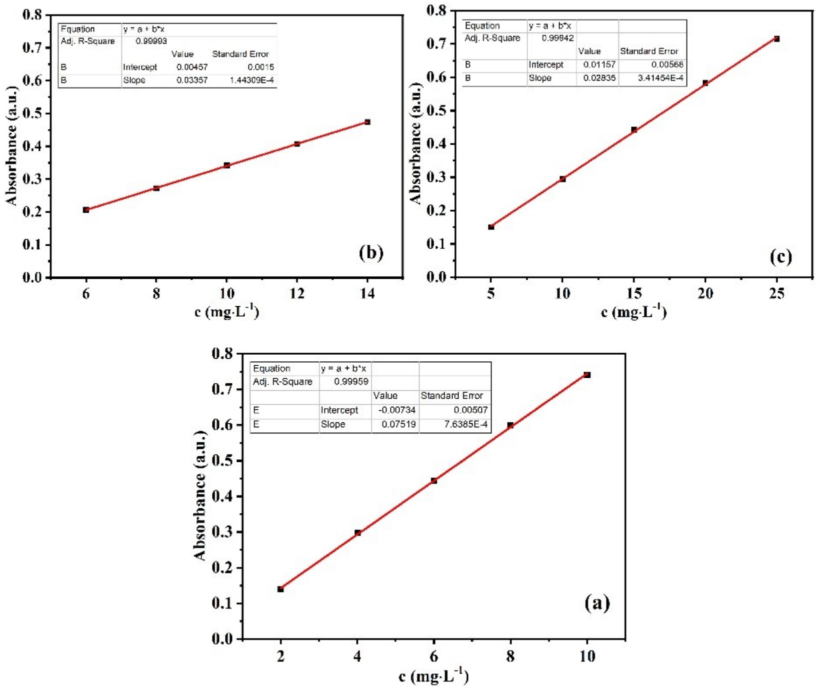

Fig.S5 A-c standard curves of EB (a), DR81(b) and MB (c).
